# P450 gene duplication and divergence led to the evolution of dual novel functions and insecticide cross-resistance in the brown planthopper *Nilaparvata lugens*

**DOI:** 10.1371/journal.pgen.1010279

**Published:** 2022-06-21

**Authors:** Ana Duarte, Adam Pym, William T. Garrood, Bartlomiej J. Troczka, Christoph T. Zimmer, T. G. Emyr Davies, Ralf Nauen, Andrias O. O’Reilly, Chris Bass

**Affiliations:** 1 College for Life and Environmental Sciences, University of Exeter, Penryn, Cornwall, United Kingdom; 2 Department of Life Sciences, Imperial College London, South Kensington Campus, London, United Kingdom; 3 Syngenta Crop Protection, Werk Stein, Schaffhauserstrasse, Stein, Switzerland; 4 Department of Biointeractions and Crop Protection, Rothamsted Research, Harpenden, United Kingdom; 5 Bayer AG, Crop Science Division, Alfred Nobel-Strasse 50, Monheim, Germany; 6 School of Biological & Environmental Sciences, Liverpool John Moores University, Liverpool, United Kingdom; University of Kentucky, UNITED STATES

## Abstract

The sustainable control of many highly damaging insect crop pests and disease vectors is threatened by the evolution of insecticide resistance. As a consequence, strategies have been developed that aim to prevent or delay resistance development by rotating or mixing insecticides with different modes of action (MoA). However, these approaches can be compromised by the emergence of mechanisms that confer cross-resistance to insecticides with different MoA. Despite the applied importance of cross-resistance, its evolutionary underpinnings remain poorly understood. Here we reveal how a single gene evolved the capacity to detoxify two structurally unrelated insecticides with different MoA. Using transgenic approaches we demonstrate that a specific variant of the cytochrome P450 CYP6ER1, previously shown to confer resistance to the neonicotinoid imidacloprid in the brown planthopper, *N*. *lugens*, also confers cross-resistance to the phenylpyrazole ethiprole. *CYP6ER1* is duplicated in resistant strains, and we show that while the acquisition of mutations in two encoded substrate recognition sites (SRS) of one of the parologs led to resistance to imidacloprid, a different set of mutations, outside of known SRS, are primarily responsible for resistance to ethiprole. Epistatic interactions between these mutations and their genetic background suggest that the evolution of dual resistance from the same gene copy involved functional trade-offs in respect to CYP6ER1 catalytic activity for ethiprole versus imidacloprid. Surprisingly, the mutations leading to ethiprole and imidacloprid resistance do not confer the ability to detoxify the insecticide fipronil, another phenylpyrazole with close structural similarity to ethiprole. Taken together, these findings reveal how gene duplication and divergence can lead to the evolution of multiple novel functions from a single gene. From an applied perspective they also demonstrate how cross-resistance to structurally unrelated insecticides can evolve, and illustrate the difficulty in predicting cross-resistance profiles mediated by metabolic mechanisms.

## Author summary

The evolution of mechanisms that confer resistance to insecticides of different mode of action (MoA) threatens to undermine the primary strategy used to manage insecticide resistance based on the rotation of insecticides of different MoA. Despite its importance, our understanding of how cross-resistance evolves remains surprisingly poor. Here, we uncover the mechanisms by which a single gene, *CYP6ER1*, encoding a cytochrome P450 enzyme, evolved resistance to two insecticides of different MoA, namely the neonicotinoid imidacloprid and the phenylpyrazole ethiprole. We have previously shown that *CYP6ER1* is duplicated in imidacloprid resistant strains of the brown planthopper, *N*. *lugens*. In the current study we demonstrate that two different sets of mutations, occurring in the same copy of *CYP6ER1*, were key to conferring the *de novo* ability to detoxify imidacloprid and ethiprole. We uncover complex interactions between these mutations and their genetic background that provide strong evidence of functional trade-offs associated with evolving dual resistance. Our findings provide fundamental and applied insights into the versatility of gene duplication in providing opportunities for functional innovation during the evolution of adaptive traits and reveal the mechanisms by which dual novel functions can arise from single genes.

## Introduction

Cross-resistance, defined as resistance to two or more pesticides conferred by the same gene, threatens to undermine strategies that aim to ensure the sustainable control of insect pests [1]. Numerous examples have been described where the evolution of resistance to one insecticide results in cross-resistance to other compounds that share the same mode of action (MoA) [2–6]. Indeed, the primary strategy of insecticide resistance management, rotation of insecticides of different MoAs, is based on the assumption that cross-resistance occurs within, but not between, MoA groups. However, while much rarer, examples of cross-resistance between insecticides of different MoA have been described [1,7,8]. Such cases typically involve metabolic resistance, i.e. the enhanced expression or activity of an enzyme that metabolises the insecticides to less toxic metabolites [7,9]. However, the mechanisms by which a single enzyme evolves the *de novo* ability to confer resistance to structurally unrelated insecticides remains unclear, and predicting cross-resistance profiles across MoA groups conferred by metabolic mechanisms is notoriously difficult.

The brown planthopper, *Nilaparvata lugens*, is an economically important pest of rice crops throughout Asia. The widespread and intensive use of insecticides against this species has led to numerous reports of resistance to many of the compounds used for control [10–12]. We previously demonstrated that resistance to one of these compounds, the neonicotinoid imidacloprid, which targets the nicotinic acetylcholine receptor (nAChR) in the insect nervous system, results from upregulation and neofunctionalization of the cytochrome P450 gene *CYP6ER1* [12–14]. Specifically, we demonstrated that while there are at least seven unique amino acid sequence variants of CYP6ER1 observed in field populations of *N*. *lugens* across Asia, just two of these, CYP6ER1vA, found in populations throughout South-East Asia, and CYP6ER1vB, found in populations in India, are highly overexpressed in resistant strains and metabolize imidacloprid [14]. Both variants are characterized by amino-acid alterations in two P450 substrate recognition sites (T318S in SRS4 in both variants and P377del in SRS5 in CYP6ER1vB, and A375del and A376G in SRS5 in CYP6ER1vA), and the introduction of these mutations into a susceptible P450 sequence is sufficient to confer resistance [14]. *CYP6ER1* is duplicated in resistant strains with individuals carrying paralogs with and without the gain-of-function mutations [14]. Intriguingly, despite numerical parity in the genome, the susceptible and mutant copies exhibit marked asymmetry in their expression with the resistant paralogs overexpressed [14]. Recent work has also demonstrated that, in addition to imidacloprid, CYP6ER1vA metabolises a range of other neonicotinoids (with the exception of dinotefuran) and the butenolide flupyradifurone, illustrating the broad cross-resistance profile of this P450 variant for insecticides that act on the nAChR [15].

Due to the growing issue of resistance to neonicotinoid insecticides in *N*. *lugens*, phenylpyrazole insecticides, such as ethiprole and fipronil, which target the gamma-aminobutyric acid (GABA)-gated chloride channel of insects, have been introduced for control [16]. However, resistance has now been reported to both compounds in field populations of *N*. *lugens* [12,17,18]. A recent study examining the sensitivity of *N*. *lugens* populations collected from across South and East Asia to imidacloprid and ethiprole found that many of the strains showed high levels of resistance to both insecticides [12]. Two of the field populations described in this study, that exhibit resistance to imidacloprid and ethiprole, were subsequently selected in the laboratory with ethiprole [19]. This led to enhanced resistance to this compound, and, intriguingly, resistance to the related phenylpyrazole fipronil [19]. Investigation of the role of target-site modification in resistance to phenylpyrazoles in these strains identified a mutation, A301S, in the RDL GABA-gated chloride channel that was shown to confer resistance to ethiprole but not fipronil [19]. However, the *in vitro* data obtained in this study on the level of resistance conferred by the A301S mutation failed to fully explain the extremely high levels of resistance seen in the ethiprole selected *N*. *lugens* strains, suggesting other mechanisms of resistance contribute to ethiprole (and fipronil) resistance [19]. Related to this, research on an ethiprole resistant strain of *N*. *lugens* collected in Thailand using inhibitors of metabolic enzymes and biochemical assessment of enzyme activities suggested that enhanced expression of P450s and esterases may contribute to resistance [20].

Motivated by the reports of *N*. *lugens* strains with resistance to imidacloprid, ethiprole and fipronil, here we investigate the molecular basis of insecticide cross-resistance, using CYP6ER1-mediated resistance in *N*. *lugens* as a case study. We ask: 1) Does CYP6ER1 confer resistance to both neonicotinoids (imidacloprid) and phenylpyrazoles (ethiprole and fipronil), and, if so, which variants confer cross-resistance? 2) Do the same mutations in SRS4 and SRS5 that were shown to be required for the evolution of catalytic activity against imidacloprid, or alternative mutations, lead to resistance to phenylpyrazoles? 3) If CYP6ER1 has evolved the capacity to detoxify insecticides belonging to different MoA groups, what was the role of gene duplication and divergence (i.e. neofunctionalization) in this process?

## Results

### Increased expression of *CYP6ER1* under selection for ethiprole resistance

In this study we exploited two multi-resistant field-collected strains of *N*. *lugens* reported previously [12,19]. The strains NLF2 and NLF7 were shown to exhibit 406-fold and 331-fold resistance to ethiprole respectively when compared to a lab susceptible strain, NLS [19]. Subsequent selection of NLF2 and NLF7 with ethiprole (resulting in the NLF2-eth and NlF7-eth strains) increased the level of resistance to > 14,000-fold in both strains [19]. NLF2 and NLF7 also exhibit 32-fold and 3-fold resistance to fipronil, and intriguingly, selection with ethiprole also resulted in increased resistance to fipronil, with NLF2-eth and NlF7-eth exhibiting approximately 860-fold resistance to this compound compared to NLS [19]. Finally, we have previously demonstrated that NLF2 and NLF7 are 283-fold and 24-fold resistant to imidacloprid respectively [14].

**Fig 1:**
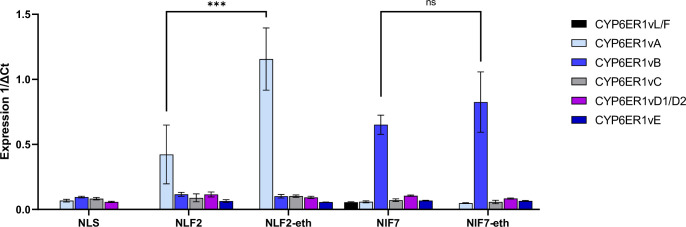
Relative expression of CYP6ER1 variants in ethiprole selected (NLF2-eth and NLF7-eth) and unselected *N*. *lugens* field strains (NLF2 and NLF7) and a reference susceptible strain (NLS), as determined by variant-specific quantitative PCR. Error bars indicate 95% confidence intervals (n = 4). Lines above bars are used to denote significant (n.s., non significant at p = < 0.05, ***, significant at p = < 0.001) differences in variant expression between selected and unselected strains as assessed by one-way ANOVA with post-hoc Tukey HSD.

Prior work has shown that NLF2 and NLF7 overexpress two different variants of *CYP6ER1*, with the former expressing *CYP6ER1vA* and the latter *CYP6ER1vB* [14]. To examine if selection of NLF2 and NLF7 with ethiprole resulted in changes in the expression of these P450 variants we used a variant-specific quantitative PCR assay. This confirmed that NLF2 and NLF7 overexpress the *CYP6ER1vA* and *CYP6ER1vB* variants respectively (**Fig 1**), consistent with previous work. Furthermore, selection of NLF2 resulted in a significant (*p* < 0.001) 2.7-fold increase in expression of *CYP6ER1vA* (**Fig 1**). In contrast, no significant increase in expression of *CYP6ER1vB* was observed in NLF7-eth compared to its unselected parental strain (**Fig 1**). The finding that selection of *N*. *lugens* with ethiprole results in enhanced expression of *CYP6ER1vA* but not *CYP6ER1vB*, suggests that overexpression of specific *CYP6ER1* variants may contribute to resistance to ethiprole, and potentially to the related phenylpyrazole fipronil.

### CYP6ER1vA confers resistance to ethiprole but not fipronil

**Fig 2:**
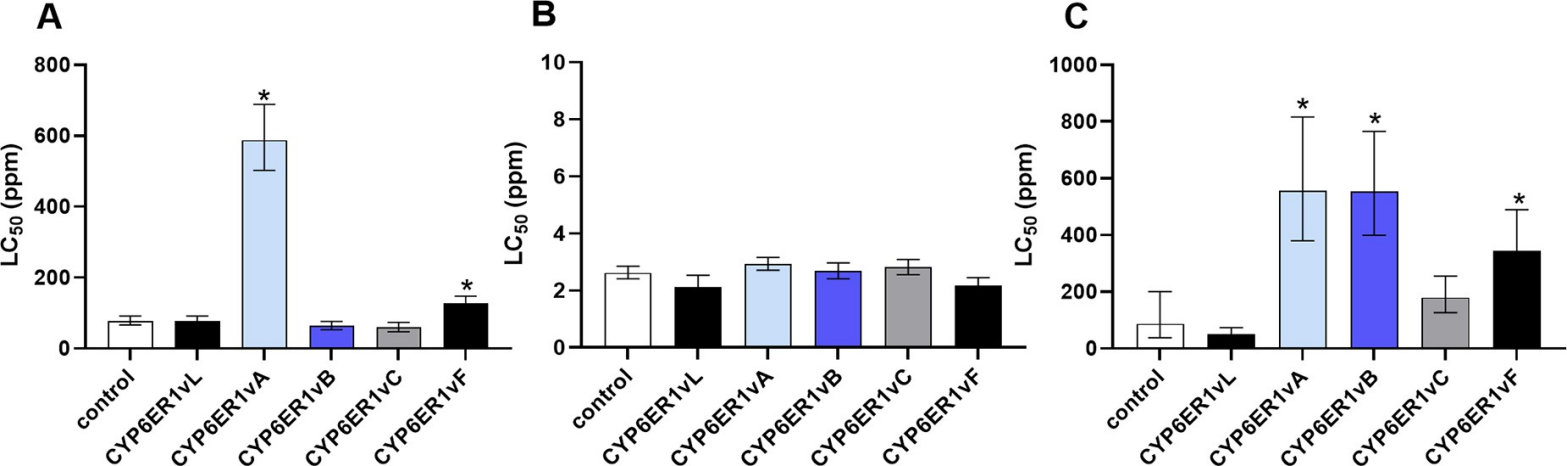
Sensitivity of transgenic strains of *Drosophila melanogaster* expressing different CYP6ER1 variants to three insecticides. Lethal Concentration 50% (LC_50_) values for each strain derived from full dose-response bioassays are displayed for A) ethiprole, B) fipronil and C) imidacloprid. Data for *D*. *melanogaster* strains expressing CYP6ER1 variants observed in field populations of *N*. *lugens* (CYP6ER1vA, B, C, F) and the lab susceptible strain NLS (CYP6ER1vL) is shown. For comparison the sensitivity of a control fly line (control) of the same genetic background but without a transgene is shown. Error bars show 95% CI (n = 4). Significant differences (p = < 0.05) in LC_50_ values of fly lines expressing CYP6ER1 variants and the fly line without a transgene, identified by *z-*tests using the compParm() function of the ’drc’ package [36], are indicated using an asterisk above relevant bars. See also S1–S3 Tables.

To examine if any of the known variants of CYP6ER1 confer resistance to ethiprole or fipronil we produced a series of transgenic *Drosophila melanogaster* strains that express each of the five primary variants of *CYP6ER1* (*CYP6ER1vA*, *B*, *C*, *L* and *F*) observed in field populations of *N*. *lugens* [14] (**S1 Fig**). When the sensitivity of these strains to ethiprole was examined in full dose-response bioassays, only the strain expressing *CYP6ER1vA* showed strong (~8-fold) and significant (*p* < 0.001) resistance to ethiprole compared to control flies of the same genetic background without the transgene, and when compared to flies expressing *CYP6ER1vL*, the ancestral *CYP6ER1* variant observed in the reference susceptible strain NLS (**Fig 2A** and **S1 Table**). To provide additional evidence that CYP6ER1vA directly metabolises ethiprole, we expressed this P450 variant *in vitro* and examined its capacity to metabolise this compound using liquid chromatography tandem mass spectrometry (LC-MS/MS). This demonstrated that CYP6ER1vA effectively metabolises ethiprole to an M+16 metabolite in the presence of NADPH (**S2 Fig**). Recovery of the M+16 metabolite from controls (preparations of recombinantly expressed CYP6ER1vA and ethiprole in the absence of NADPH) was below the limit of detection.

In contrast, bioassays with fipronil revealed changes in tolerance of <1.2-fold for any of the *CYP6ER1* expressing lines to this compound compared to flies without the transgene, and all transgenic fly strains exhibited similar levels of fipronil sensitivity (**Fig 2B** and **S2 Table**). Finally, although previous work has demonstrated that two variants of CYP6ER1 (CYP6ER1vA and CYP6ER1vB) confer resistance to imidacloprid *in vitro* [14], we also tested our transgenic fly lines against imidacloprid to enable direct comparison with bioassay data for fipronil and ethiprole. This corroborated the *in vitro* results reported previously by demonstrating that both *CYP6ER1vA* and *CYP6ER1vB* confer significant (*p* < 0.001) resistance (6.4-fold compared to flies without the transgene) to imidacloprid *in vivo* (**Fig 2C** and **S3 Table**). Lower (4-fold compared to flies without the transgene), but significant (*p* < 0.02), levels of imidacloprid resistance were also observed in flies expressing the CYP6ER1vF variant (**Fig 2C** and **S3 Table**). Taken together, these results demonstrate for the first time that, in addition to conferring resistance to imidacloprid, certain variants of CYP6ER1 (i.e. CYP6ER1vA) also confer strong resistance to ethiprole, but not to the related phenylpyrazole fipronil.

### Do the mutations in *CYP6ER1vA* that lead to resistance to imidacloprid result in cross-resistance to ethiprole?

**Fig 3:**
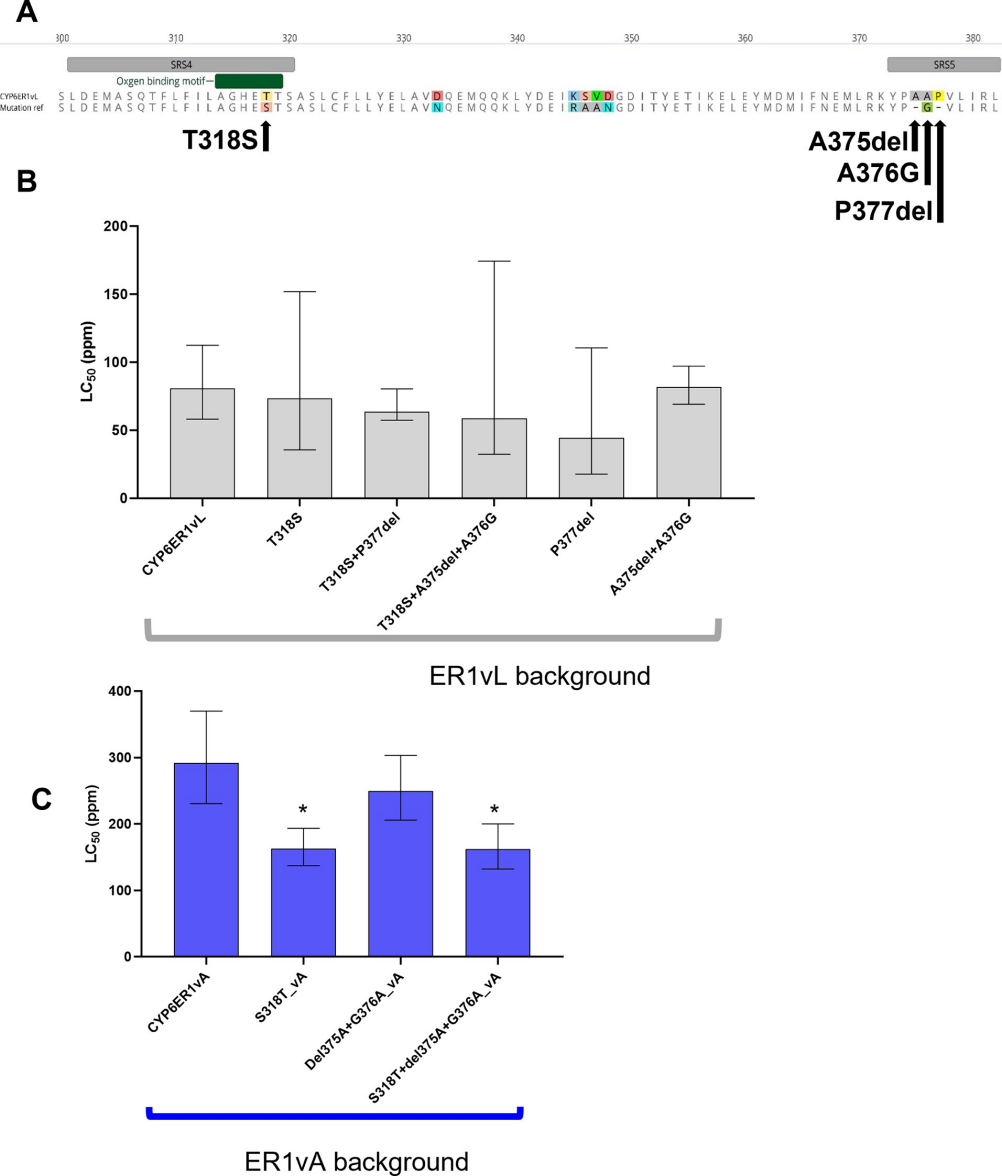
Sensitivity of transgenic strains of *Drosophila melanogaster* expressing mutated CYP6ER1 variants to ethiprole. A) Amino acid alignment of CYP6ER1vL and a notional CYP6ER1 sequence displaying the nature and position of mutations previously implicated in imidacloprid resistance in the SRS4 and SRS5 of CYP6ER1vA and CYP6ER1vB. B, C) Lethal Concentration 50% (LC_50_) values derived from full dose-response bioassays are displayed for *D*. *melanogaster* strains expressing CYP6ER1vL variants to which mutations previously implicated in imidacloprid resistance in the SRS4 and SRS5 of CYP6ER1vA were added (B), or CYP6ER1vA to which mutations previously implicated in imidacloprid resistance were removed (C). For comparison LC_50_ values derived from flies expressing the wildtype versions of CYP6ER1vA and CYP6ER1vL are shown. Error bars show 95% CI (n = 4). Significant differences (p = < 0.05) in LC_50_ values of fly lines expressing CYP6ER1 mutated variants and the respective wildtype fly line (CYP6ER1vA or CYP6ER1vL), identified by *z-*tests using the compParm() function of the ’drc’ package [34], are indicated using an asterisk above relevant bars. See also S4 and S5 Tables.

CYP6ER1vA and CYP6ER1vB carry mutations in two P450 substrate-recognition sites (SRS4 and SRS5) that were previously implicated in the metabolism of imidacloprid [14]. The first of these mutations, shared by vA and vB, results in an amino acid substitution at SRS4 at position 318, in which a threonine (in all other variants) is replaced by a serine (T318S). Significantly, this occurs at a highly conserved position in a P450 signature sequence [A/G]GX[E/D]T[T/S] in helix I, known as the oxygen-binding motif. In SRS5, vA carries mutations that are unique to this variant that result in the deletion of an alanine (Ala375), followed by an alanine to glycine substitution at position 376 (A375del+A376G), in vB mutations result in the deletion of a proline at position 377 (P377del) (**Figs 3A and S1**). To understand if these mutations play any role in ethiprole metabolism, we performed ethiprole bioassays on transgenic *D*. *melanogaster* strains expressing mutated versions of *CYP6ER1*, into which mutations had been either introduced into a vL gene sequence (the ancestral *CYP6ER1* variant observed in the reference susceptible strain NLS) or replaced by wildtype (vL) in a vA sequence (one of the two CYP6ER1 variants previously shown to metabolise imidacloprid) (**Fig 3A**). Surprisingly, none of these mutations, when introduced in a vL sequence, conferred ethiprole resistance in transgenic *D*. *melanogaster* (*p* = 0.22–0.95, **Fig 3B** and **S4 Table**).

Replacing the serine at position 318 by the wildtype threonine in the vA sequence slightly reduced resistance to ethiprole (*p* < 0.001, RR = 0.6 compared to vA, **Fig 3C** and **S5 Table**). Restoring the wildtype double alanine at positions 375–376, in a vA sequence, resulted in no significant loss of resistance. Restoring the vL amino acids at all three positions in a vA sequence, caused a modest but statistically significant reduction in resistance (*p* < 0.001, RR = 0.6, **Fig 3C** and **S5 Table**). Overall, these results demonstrate that amino acid variation in SRSs 4 and 5 does not, on its own, determine the metabolism of ethiprole by CYP6ER1, although it may contribute to it.

### Are mutations outside SRS 4 and 5 involved in resistance to ethiprole?

**Fig 4:**
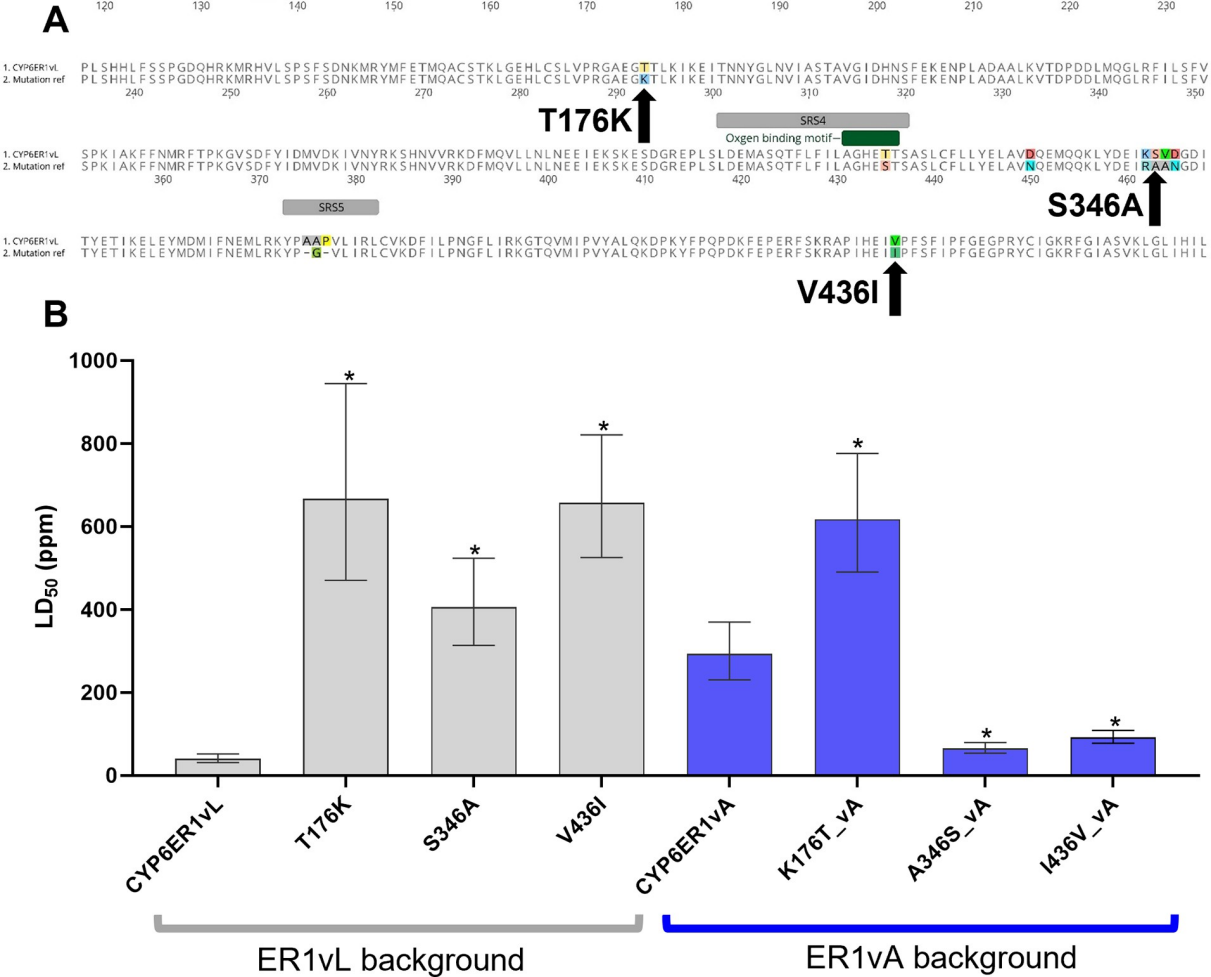
Sensitivity of transgenic strains of *Drosophila melanogaster* expressing mutated CYP6ER1 variants to ethiprole. A) Amino acid alignment of CYP6ER1vL and a notional CYP6ER1 sequence displaying the nature and position of three mutations found in *CYP6ER1vA* outside of SRS4 and SRS5. B) Lethal Concentration 50% (LC_50_) values derived from full dose-response bioassays are displayed for *D*. *melanogaster* strains expressing *CYP6ER1vL* variants to which three mutations found in *CYP6ER1vA* outside of SRS4 and SRS5 were added, or *CYP6ER1vA* to which the three mutations were removed. For comparison LC_50_ values derived from flies expressing the wildtype versions of CYP6ER1vA and CYP6ER1vL are shown. Error bars show 95% CI (n = 4). Significant differences (p = < 0.05) in LC_50_ values of fly lines expressing CYP6ER1 mutated variants and the respective wildtype fly line (CYP6ER1vA or CYP6ER1vL), identified by *z-*tests using the compParm() function of the ’drc’ package [36], are indicated using an asterisk above relevant bars. See also S6 Table.

Given the finding that the mutations leading to resistance to imidacloprid, play, at best, a minor role in resistance to ethiprole we asked if alternative amino acid substitutions in CYP6ER1vA are more important determinants of ethiprole metabolism. CYP6ER1vA exhibits three amino acid substitutions in the regions outside of SRS 4 and 5 (**Figs 4A** and **S1**) that are unique in this variant relative to the other variants tested here, and are thus candidates for a role in resistance to ethiprole. Interestingly, these variants are all outside of the six known P450 substrate recognition sites, which is why they were not previously considered strong candidates for functional investigation of imidacloprid resistance. Specifically, at amino acid position 176, a threonine in all other variants is replaced by a lysine in vA (T176K), at position 346 a serine is replaced by an alanine (S346A), and at position 436 a valine is substituted by an isoleucine in vA (V436I) (**Fig 4A**). In the case of the latter, it is important to note that V436I is present in other variants of CYP6ER1 (vD and vE), which are not included in this study, and are present at low frequencies in imidacloprid-resistant populations (**S1 Fig**) [14]. To investigate the role of these mutations in ethiprole resistance we created additional transgenic fly lines expressing mutated versions of CYP6ER1, into which the mutations were either introduced into a vL gene sequence (the ancestral *CYP6ER1* variant observed in the reference susceptible strain NLS) or replaced by wildtype (vL) in a vA sequence. Insecticide bioassays of these fly lines (**Fig 4B** and **S6 Table**) revealed that adding T176K to vL results in significant (*p* < 0.001) and strong resistance to ethiprole. Remarkably, the level of resistance exhibited by the fly line expressing this version of CYP6ER1 was even greater than that of CYP6ER1vA tested in the same bioassay (RR = 16.54 versus 7.25 in vA). Surprisingly, however, restoring the wildtype amino acid at this position (K176T) in a vA sequence did not result in a reduction in resistance, with resistance actually increasing relative to CYP6ER1vA (*p* < 0.001, RR = 2.1). Introducing the S346A replacement in vL also resulted in significant (*p* < 0.001) and strong resistance to ethiprole (RR = 10.06), and, when removed from vA, a significant (*p* < 0.001) loss of resistance was observed (RR = 0.2). Finally, adding V436I to vL, resulted in significant (*p* < 0.001) and strong resistance to ethiprole (RR = 16.30), and when removed from vA, led to a significant (*p* < 0.001) reduction in resistance (RR = 0.3).

These findings demonstrate that alternative mutations to those implicated in imidacloprid resistance, that occur outside of known P450 SRSs, confer resistance to ethiprole.

### Do the mutations outside SRS 4 and 5 play a role in imidacloprid resistance?

**Fig 5:**
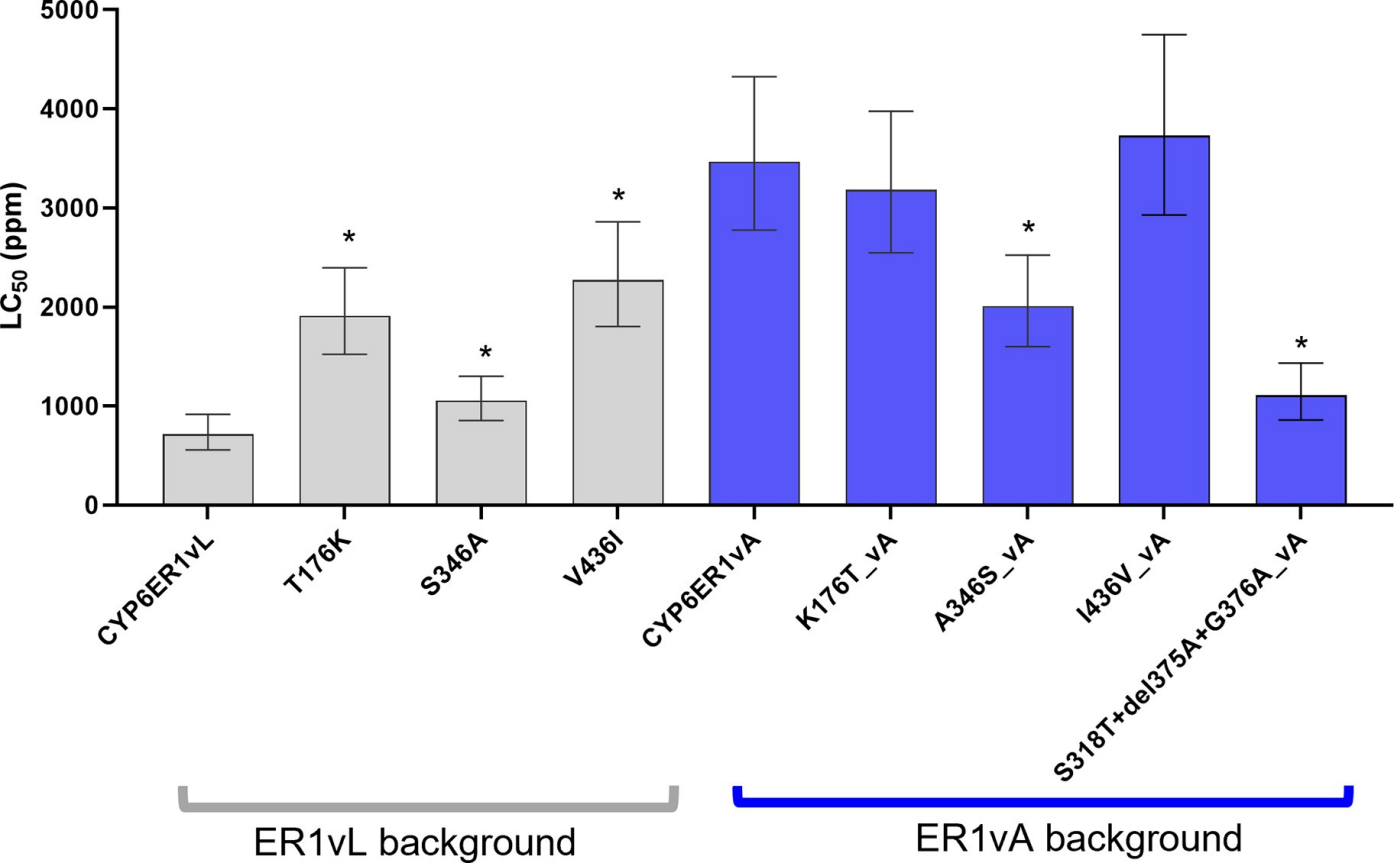
Sensitivity of transgenic strains of *Drosophila melanogaster* expressing mutated CYP6ER1 variants to imidacloprid. Lethal Concentration 50% (LC_50_) values derived from full dose-response bioassays are displayed for *D*. *melanogaster* strains expressing *CYP6ER1vL* variants to which mutations found in *CYP6ER1vA* were added, or *CYP6ER1vA* to which mutations were removed. For comparison LC_50_ values derived from flies expressing the wildtype versions of CYP6ER1vA and CYP6ER1vL are shown. Error bars show 95% CI (n = 4). Significant differences (p = < 0.05) in LC_50_ values of fly lines expressing CYP6ER1 mutated variants and the respective wildtype fly line (CYP6ER1vA or CYP6ER1vL), identified by *z-*tests using the compParm() function of the ’drc’ package [36], are indicated using an asterisk above relevant bars. See also S7 Table.

To examine if the mutations associated with resistance to ethiprole are associated with cross-resistance to the structurally unrelated insecticide imidacloprid, the sensitivity of transgenic flies expressing T176K, V436I and S436A in a vL background, and the reverse substitutions in a vA background (i.e., K176T, A346S and I436V), to this compound was examined. Two of the mutations, T176K and V436I, induced modest but statistically significant (*p* < 0.001, ~3-fold) levels of resistance to imidacloprid when inserted in a vL gene sequence. However, when these two mutations were removed from vA (i.e., K176T and I436V in a vA background) resistance to imidacloprid was not significantly affected (*p =* 0.6 and 0.7 respectively, **Fig 5** and **S7 Table**). In the case of S346A very low (1.5-fold) but statistically significant (*p* < 0.03) resistance to imidacloprid was observed when inserted in a vL gene sequence and a slight (RR = 0.6) but statistically significant (*p* < 0.001) reduction in resistance was observed for A346S. These findings were in contrast to the result of changing the mutations previously implicated in imidacloprid resistance in CYP6ER1vA located in SRSs 4 and 5. When these were changed to wildtype (S318T+del375A+G376A) a marked loss of imidacloprid resistance (*p* < 0.001, RR = 0.3) was observed (**Fig 5** and **S7 Table**). Taken together these findings suggest the mutations involved in conferring resistance to ethiprole are not critical determinants of imidacloprid resistance.

### Location of T176K, S346A and V436I in a model of CYP6ER1vA

A 3-dimensional model of CYP6ER1vA was generated in order to assess the effects that mutations may have on the structure of the enzyme. The molecular model was generated using AlphaFold2 prediction software [21] and the model quality was verified using ERRAT [22] and PROCHECK [23]. The ERRAT score of 93.75 indicates a high-quality model and a Ramachandran plot shows only one amino acid in a disallowed region (**S3 Fig**).

**Fig 6:**
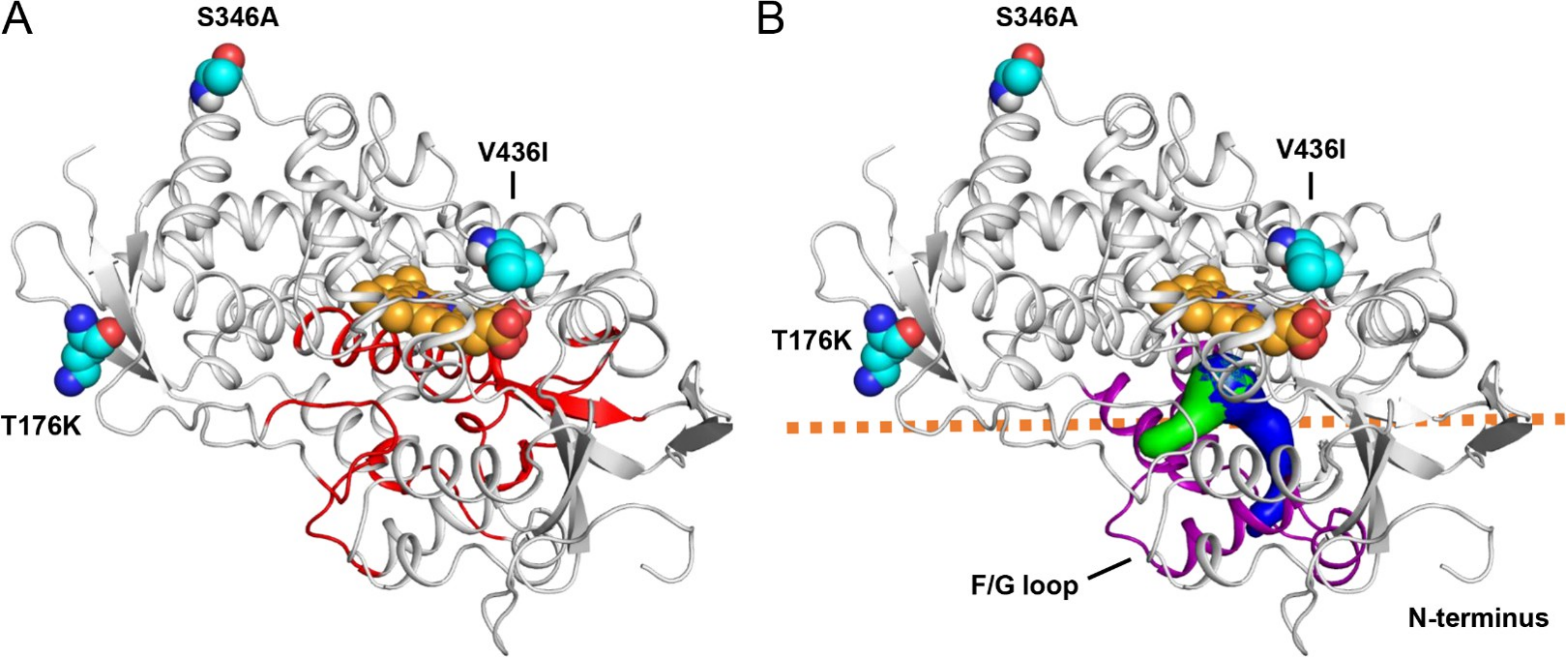
**CYP6ER1vA model showing sequence variants (cyan) and heme (orange).** (A) SRS1-6 regions are coloured red. (B) Access tunnels leading to the active site are shown in surface format. The predicted membrane-solution interface is depicted with orange dashes. The F/G loop is coloured purple.

**Fig 6** shows that the T176K, S346A and V436I mutations are located on different loop regions on the surface of the enzyme. The CYPED database [24] was interrogated to help map the mutations in relation to structurally-conserved regions: T176K is located on the loop between α-helices D and E, S346A is the C-terminal residue of α-helix J whereas V436I is located on the loop between the meander loop and the Cys pocket. As mentioned above, these mutations are not found in the SRS regions in the primary sequence (**Fig 4A**), and the model shows that they are also distant from the SRS regions in 3-dimensional space (**Fig 6A**), and so are not positioned to be direct binding determinants for substrates. CAVER software [25] was then used to detect and plot access pathways to the active site to determine if the mutations are lining these tunnels; **Fig 6B** shows two of the most prominent tunnels. The S346A and V436I residues are located on the proximal side of the enzyme and are not in a position where their substitution would directly reshape a substrate access pathway. Similarly, although T176K is found on the distal side of the enzyme, it is also too distant to directly affect an access pathway.

As eukaryotic CYP enzymes are partially immersed in a membrane environment [26], we sought to determine the position of the mutations relative to the lipid bilayer. **Fig 6B** shows a prediction for CYP6ER1vA immersed in a membrane based on a similar pose described by Šrejber et al [26] for the CYP3A4 enzyme; common features include the F/G loop and N-terminal region of both enzymes buried in the lipid bilayer. While S346A and V436I residues are distant from the membrane-solution interface, the T176K residue is near the membrane surface (**Fig 6B**). The T176K mutation substitutes a polar side chain with a positively-charged side chain, which could introduce an electrostatic interaction with negatively-charged head-groups of membrane lipid molecules. If this attractive interaction results in a change in the position and orientation of CYP6ER1vA relative to the membrane, then access pathways to the active site could also be repositioned. For example, an access pathway that is buried in the lipid bilayer could get repositioned nearer the surface in the mutant enzyme and make it more accessible to hydrophilic compounds.

### The different insecticidal effect of structurally-similar fiproles on transgenic flies

**Fig 7:**
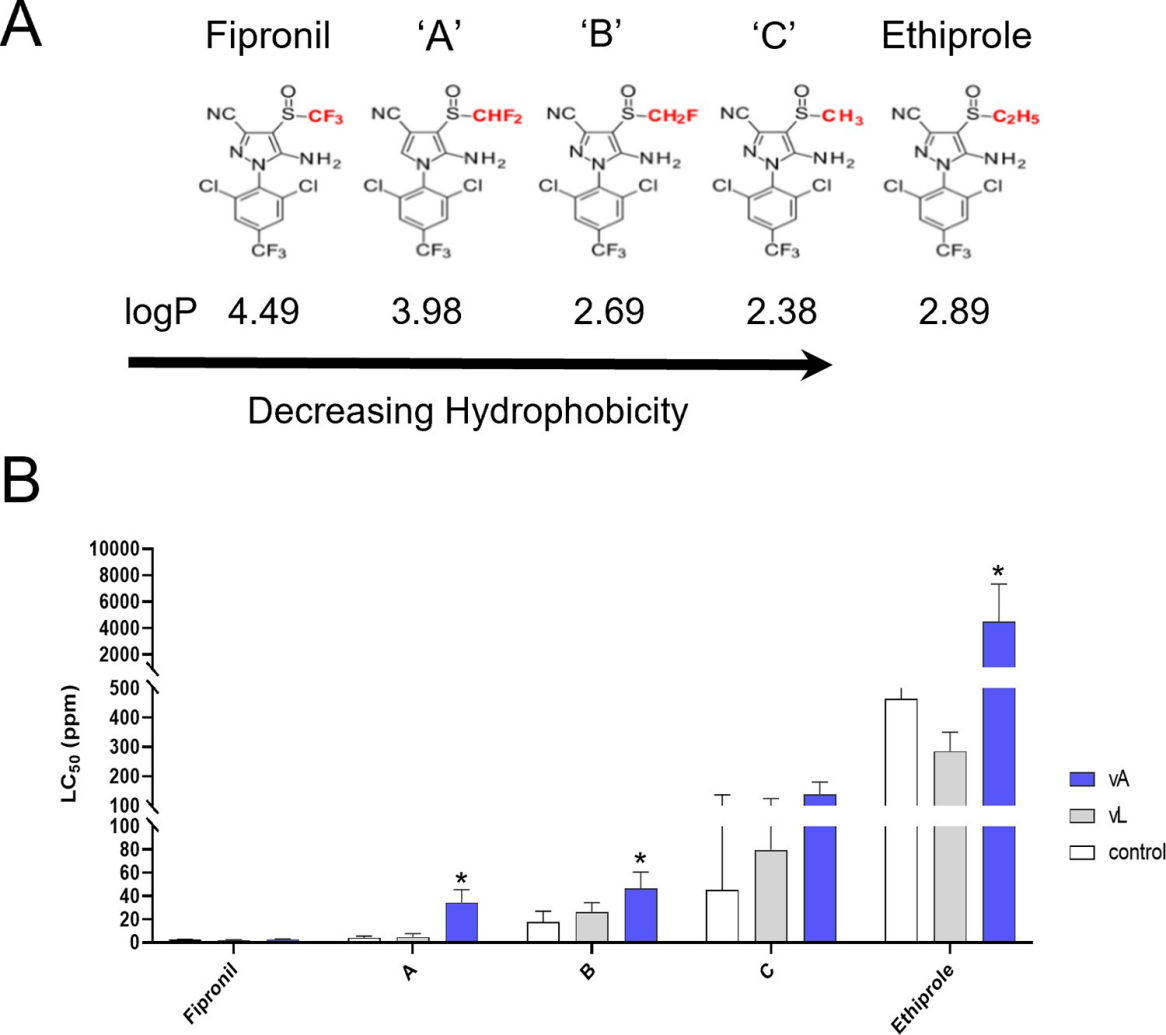
Substrate lipophilicity and relationship to insecticidal efficacy. A) Atoms that are substituted in the chemical structures of fipronil, ethiprole and compounds of intermediate halogenation are coloured red. B) Lethal Concentration 50% (LC_50_) values derived from full dose-response bioassays are displayed for *D*. *melanogaster* strains expressing CYP6ER1vA, CYP6ER1vL or no transgene for the compounds fipronil, ethiprole and their intermediates. Error bars show 95% CI (n = 4). Significant differences (p = < 0.05) in LC_50_ values of fly lines expressing CYP6ER1vA and the no transgene control, identified by *z-*tests using the compParm() function of the ’drc’ package [36], are indicated using an asterisk above relevant bars. See also S8 Table.

To assess if substrate lipophilicity is a factor in metabolism by CYP6ER1 enzymes, we synthesised a series of fiproles that represent intermediate structures between fipronil and ethiprole (**Fig 7A**). We selected three halo-methylsulfinyl derivatives with intermediate levels of halogenation that we call A, B, and C, where halogenation decreases from A to C. The calculated logP value of these compounds decreases with each successive fluorine-to-hydrogen atom substitution (**Fig 7A**). Fipronil has the largest value (logP = 4.49) and is therefore predicted to partition more readily into the lipid bilayer compared to the other compounds, whereas compound C lacking fluorine atoms in this target sulfinyl group has the lowest logP value of 2.38. Ethiprole has a logP value of 2.89 but differs from the other compounds as it has an additional methyl group to make the ethylsulfinyl moiety.

When the sensitivity of transgenic flies expressing CYP6ER1vA, CYP6ER1vL or no transgene was examined, a gradual decrease in toxicity against all the fly lines tested was observed moving from fipronil and ethiprole (**Fig 7B** and **S8 Table**). This trend was greatest for flies expressing CYP6ER1vA, which exhibited a significant (*p* < 0.001) degree of resistance to all the compounds tested except for fipronil and compound C (*p* > 0.05), ranging from 2.6-fold (compound B) to 9.7-fold (ethiprole) when compared to control flies that did not express a transgene. However, a linear increase between a decreasing level of halogenation and resistance was not observed, thus while this data demonstrates that the single substituent ethylsulfinyl of ethiprole vs trifluoromethylsulfinyl of fipronil strongly influences the ability of CYP6ER1vA to metabolize phenylpyrazoles, the level of halogenation of this moiety *per se* is not the sole determinant of metabolic fate.

## Discussion

Here we present an example of the evolution of dual novel functions by gene duplication and divergence that allowed a single gene to encode the ability to metabolise two structurally unrelated insecticides. Remarkably, mutations in CYP6ER1vA that were previously shown to be critically important determinants of imidacloprid resistance [14], do not seem to play a key role in resistance to ethiprole. Rather, we identify three novel mutations in CYP6ER1vA, outside of the six known substrate-recognition sites, that promote strong resistance to ethiprole *in vivo*. Our findings provide fundamental insights into i) the versatility of gene duplication in providing opportunities for functional innovation during the evolution of adaptive traits, ii) constraints and trade-offs in the evolution of dual novel functions, iii) the role of amino acids outside known SRSs on the substrate specificity of P450s, and, iv) the molecular mechanisms of insecticide cross-resistance. These topics, and their applied implications for the control of highly damaging insect pests, are discussed below.

Our data demonstrate that the P450 CYP6ER1, previously implicated in imidacloprid resistance [14], also confers resistance to another widely used, but structurally unrelated, insecticide, ethiprole. More specifically we show that only one (*CYP6ER1vA*) of the five primary variants of *CYP6ER1* observed in field populations of *N*. *lugens* confers strong resistance to ethiprole. This finding explains why *CYP6ER1* expression only increased under ethiprole selection in a *N*. *lugens* population (NLF2) where vA was the predominant variant, and not in a strain that predominantly expressed the vB variant. Together, *CYP6ER1vA* and *CYP6ER1vB* are the two most highly expressed variants observed in insecticide resistant field populations of *N*. *lugens* across Asia, with the former found in populations throughout southeast Asia, but the latter showing a more restricted distribution to India [14]. The ability of CYP6ER1vA to metabolise two of the most widely used insecticides in Asia may explain why it is the most widespread, and highly expressed CYP6ER1 variant observed in *N*. *lugens* populations worldwide.

As detailed in the introduction, *CYP6ER1* is duplicated in insecticide resistant *N*. *lugens* strains, and individuals of the ethiprole and imidacloprid resistant NLF2 strain carry one copy with the gain-of-function mutations (*CYP6ER1vA*) and one without (*CYP6ER1vC*) [14]. We show that transgenic flies expressing *CYP6ER1vC*, or the ancestral variant (*CYP6ER1vL*) present as a single copy in insecticide susceptible *N*. *lugens*, exhibit no resistance to ethiprole. Thus, the evolution of the novel, selectively beneficial function of the mutant copy of *CYP6ER1* (i.e. ethiprole metabolism), which was not present in the ancestral gene, represents a second example of neofunctionalization of this P450 gene. This evolution of dual novel functions (imidacloprid and ethiprole resistance) from a single gene illustrates the versatility of gene duplication in providing opportunities for functional innovation. In the case of CYP6ER1, recent work has shown that this P450 is important for organismal fitness [27], thus gene duplication was likely key to creating redundancy and freeing the second gene copy from the functional constraints associated with maintaining its endogenous function, allowing continued divergence from the ancestral single copy gene.

Insecticide bioassays of transgenic flies expressing CYP6ER1 carrying different amino acid alterations revealed evidence of complex interactions between the mutations associated with ethiprole resistance and the genetic background into which they are inserted. When added to a susceptible vL background, each mutation on its own was able to promote strong resistance, to levels comparable to, or higher than, the natural variant vA. Furthermore, when each mutation was removed from a vA background, resistance declined in two cases (A346S and I436V). If each mutation had an independent effect, we would expect resistance to be promoted when each mutation alone was present in a vL sequence, and that resistance would not revert to the wildtype phenotype if each single mutation was removed from a vA background, because the other two mutations would still be present. Rather, our data reveal strong epistatic interactions between the mutations that influence the presence and magnitude of resistance. While further research is required to unravel these interactions, the pattern of mutations observed in CYP6ER1vA, and the phenotypes they confer individually and in combination, likely reflect the evolutionary constraints imposed by the requirement for this enzyme to metabolise two structurally unrelated insecticides. Resistance to ethiprole evolved after resistance to imidacloprid, due to the fact that imidacloprid use predates ethiprole use in field populations of *N*. *lugens* [12]. While the mutations associated with ethiprole resistance induced low levels of imidacloprid resistance in a vL background, their removal from vA had no effect on imidacloprid resistance in two out of the three cases, in contrast to the dramatic loss of resistance observed when the mutations in SRSs 4 and 5 were removed. Thus, preserving the mutations in SRS 4 and 5 that affect substrate specificity to imidacloprid, while additional mutations conferring resistance to ethiprole emerged, was likely key to allowing CYP6ER1 to metabolise both imidacloprid and ethiprole. This may also explain, in part, why mutations promoting strong resistance to ethiprole are located outside known P450 substrate recognition sites.

Our data also provide an indication that preserving CYP6ER1vA catalytic activity against imidacloprid may have constrained the evolution of optimal catalytic activity for ethiprole. Specifically, addition of the novel mutations conferring ethiprole resistance to a susceptible vL background (i.e. without the mutations associated with imidacloprid resistance in SRSs 4 and 5) led to higher levels of resistance than exhibited by CYP6ER1vA (where the mutations in, and outside of, SRSs 4 and 5 are found in combination) in two of the three cases. Thus, although gene duplication released *CYP6ER1* from the constraints imposed by the requirement to preserve its native function, the evolution of dual functions, each likely characterized by distinct fitness optima, from the new gene copy likely led to new functional trade-offs.

Despite numerical parity of *CYP6ER1vA* and *CYP6ER1vC* in the genome of resistant *N*. *lugens*, the susceptible and mutant copies exhibit marked asymmetry in their expression with the resistant paralog overexpressed [14]. Our previous work demonstrated that this is associated with an extended region of novel sequence upstream of *CYP6ER1vA* that provides *cis*-acting elements that result in its increased expression [14]. The increased expression of this variant under selection seen in our study, suggests that overexpression of this P450 acts in concert with the functional divergence we describe above to confer strong resistance. Furthermore, the marked overexpression of *CYP6ER1vA* relative to the ancestral gene copy may have also buffered against any negative impact resulting from the functional trade-offs associated with the evolution of dual resistance to imidacloprid and ethiprole.

Surprisingly, while CYP6ER1vA confers resistance to the structurally unrelated insecticides ethiprole and imidacloprid, which belong to completely different mode of action groups, it confers no resistance to fipronil, despite the marked chemical similarity between the two phenylpyrazoles. Ethiprole has a larger ethylsulfinyl moiety and so one possibility is that the S346I, V436I or T176K mutations allosterically modify access pathway(s) to enable ethiprole ingress. However, given the peripheral location of these residues, the inherent flexibility of P450 enzymes and their plasticity upon ligand binding [28], it is difficult to make predictions of specific structural changes that each mutation may induce. Another intriguing possibility is that the mutations affect CYP6ER1vA interactions with the membrane, particularly as T176K is located in proximity to the membrane surface and the introduced positively-charged side-chain could introduce an attractive electrostatic interaction with anionic lipid molecules.

Molecular dynamics simulations with CYP3A4 found that interactions with negatively-charged lipid headgroups affected orientation of this protein with respect to the membrane and it was proposed that enzymatic activity may consequently be affected by the opening, closing or otherwise repositioning of substrate-access tunnels [29]. If CYP6ER1vA is similarly reorientated, then this mechanism could explain our findings with CYP6ER1vA-expressing flies, which exhibited no resistance towards fipronil but a trend of increased resistance as the lipophilicity of three structurally-similar fipronil analogues decreased. The repositioning of an access pathway closer to the membrane surface could make it more accessible to these compounds, whereas the more lipophilic fipronil molecules may not reach this access tunnel if they are buried deeper within the membrane. This mechanism could also be relevant for ethiprole, although curiously the reverse K176T substitution of CYP6ER1vA did not lead to any loss of resistance to this compound, suggesting the involvement of more complex mechanism(s). Regardless, our findings provide a compelling example of the importance of residues outside the six classical substrate recognition sites in influencing the capacity of P450s to metabolise insecticides.

Finally, further studies are necessary to understand the molecular mechanisms of ethiprole resistance in populations where *CYP6ER1vA* is absent or rare. For example, those expressing *CYP6ER1vB*, such as the NLF7 strain described in this study.

In summary, our data, in combination with previous research, reveal the molecular mechanisms underpinning the evolution of insecticide cross-resistance. More broadly, these findings illustrate the power of gene duplication and subsequent divergence in providing a rich source of genetic variation on which selection can act, and demonstrate how this can lead to the evolution of multiple novel functions from a single gene. Conversely, our data also illustrate the difficulties in predicting the cross-resistance spectrum conferred by metabolic resistance mechanisms.

## Methods

### N. lugens strains

All *N*. *lugens* strains were maintained in the laboratory on whole rice plants. NLS is a long-term laboratory maintained strain that has been previously used as an insecticide susceptible reference strain. NLF2 and NLF7 were collected from the field in South Vietnam, November 2010, and East Godavari District, Andhra Pradesh, India, February 2012, respectively, and brought to the laboratory. NLF2 and NLF7 were shown to exhibit high levels of resistance to ethiprole in a previous study where they were named NL33 and NL55 [19]. To further select for ethiprole resistance, the NLF2 and NLF7 strains were reared on rice plants sprayed with increasing concentrations of ethiprole (from 7 to 100 mg L^-1^) for 5 generations, after which they were reared on rice plants sprayed with 100 mg L^-1^ every other generation to generate the NLF2-eth and NLF7-eth strains [19]. Control cultures of NLF2 and NLF7 were maintained on untreated rice plants.

### Quantitative PCR

Allele-specific quantitative PCR analysis of the expression of different *CYP6ER1* variants was performed using the primers detailed in **S9 Table** and the methods described previously [14]. Briefly, PCR reactions (20 μl) contained 10 ng of cDNA, 10 μl of SYBR Green JumpStart Taq Readymix (Sigma), and 0.25 μM of each primer. Samples were run on a Rotor-Gene 6000 (Corbett Research) using temperature cycling conditions of: 2 min at 95°C followed by 40 cycles of 95°C for 15 s, 57°C for 15 s and 72°C for 20 s. A final melt-curve step was included post-PCR (ramping from 72°C–95°C by 1°C every 5 s) to check for nonspecific amplification. The efficiency of PCR for each primer pair was assessed using a serial dilution of 100 ng to 0.01 ng of cDNA. Each quantitative RT-PCR experiment consisted of three independent biological replicates with two technical replicates for each. Data were analyzed according to the ΔC_T_ method [30] using the geometric mean of two reference genes (actin, GenBank: KU196668.1, and α2-tubulin, GenBank: FJ810204.1) for normalization [31].

### Molecular modelling of CYP6ER1

A 3-dimensional model of the CYP6ER1vA enzyme with T176K, S346A and V436I mutations was generated using the AlphaFold2 structure prediction software [21]. The CYP6ER1vA primary sequence was submitted to the ColabFold web interface [32] and default options were selected to run AlphaFold2, with the multiple sequence alignment step performed using MMseqs2 [33]. The generated models lacked the heme group and so coordinates for this cofactor were retrieved from the 2.05 Å-resolution crystal structure of human microsomal P450 3A4 (PDB code 1TQN). Amino acids within 4.5 Å of the 3A4 heme group were superimposed upon the equivalent amino acids of the top-ranking CYP6ER1vA model using SWISS-PdbViewer [34]. The heme coordinates were copied to the CYP6ER1vA structure and this holoenzyme was subjected to 100 steps of conjugate-gradient energy minimization in SWISS-PdbViewer. The quality and stereochemical soundness of the model was assessed using ERRAT [22] and PROCHECK [23] as implemented by the Structural Analysis and Verification Server (https://saves.mbi.ucla.edu/). Access pathways to the active site of the CYP6ER1vA model were detected using CAVER 3.0 [25] with a probe radius of 0.9 Å, shell radius of 2 Å and a shell depth of 3 Å. Figures were produced using PyMOL (DeLano Scientific, San Carlos, CA, USA).

MarvinSketch (v19.22) of the ChemAxon suite (http://www.chemaxon.com) was used to both generate 3-dimensional structures of fipronil, ethiprole and three halo-methylsulfinyl derivatives compounds and to calculate for each compound the log of partition coefficient (logP), where P is the octanol-water partition coefficient.

### Transgenic expression of natural and mutant CYP6ER1 variants

CYP6ER1 variants (**S1 Fig**) were synthesized and provided in the pUASTattB40 plasmid (Geneart, CA, USA). Using the PhiC31 system, clones were transformed into the germline of a *D*. *melanogaster* strain carrying the attP40 docking site on chromosome 2 [“y1w67c23; P attP40 25C6,” “1;2”] (Cambridge Fly Facility). The transgenic lines obtained were balanced and the integration of genes confirmed by PCR and sequencing (Eurofins Genomics). We crossed virgin females of the Act5C-GAL4 driver strain (Bloomington Stock Center) with UAS-gene-of-interest males (our detailed methodology has been published previously [35]). As a control, we crossed females from the driver strain with males from a control line possessing the genetic background into which the pUASTattB40 plasmid was inserted. Overexpression of the inserted genes in the F1 offspring, relative to parental lines, was confirmed by qPCR (**S4** and **S5 Figs**) using the methods described above and the primers shown in **S9 Table**. Adult female flies expressing the gene of interest were used for bioassays to assess their resistance to ethiprole, fipronil and imidacloprid. A range of insecticide concentrations were overlaid onto 1.5% agar containing 1% sucrose in standard Drosophila vials and allowed to dry overnight at room temperature. Twenty adult flies (two to five days post eclosion) were then added to each vial and mortality assessed after 48 h. Control mortality was assessed using tubes containing agarose/sucrose minus insecticide. A minimum of four replicates were carried out for each concentration. Lethal concentration (LC_50_ values) and 95% confidence intervals (CIs) were calculated with 2 parameter log-logistic models using the R package ‘drc’ [36]. Resistance ratios (RR) and their 95% CI were calculated with respect to the susceptible strain vL within each bioassay, using the function EDcomp() in ‘drc’. *Z*-tests using compParm() function in ‘drc’ were used to identify statistically significant difference in the LC_50_ values of different strains.

### Heterologous expression of CYP6ER1vA

CYP6ER1vA (GenBank accession number MF970458) and *M*. *domestica* NADPH-dependent cytochrome P450 reductase (CPR) (GenBank accession number Q07994) were codon optimized for expression in lepidopteran cells and obtained by gene synthesis (GeneArt, CA, USA) in the pDEST8 expression vector (Invitrogen). The PFastbac1 vector with no insert DNA was used to produce a control virus. The recombinant baculovirus DNA was constructed and transfected into Sf9 cells using the Bac-to-Bac baculovirus expression system (Invitrogen) according to the manufacturer’s instructions. The titer of the recombinant virus was determined following the protocols of the supplier. High Five cells grown to a density of 2 × 10^6^ cells/mL^-1^ were co-infected with recombinant baculoviruses containing P450 and CPR at various MOI (multiplicity of infection) ratios to identify the best conditions. Control cells were co-infected with the baculovirus containing vector with no insert (ctrl-virus) and the recombinant baculovirus expressing CPR using the same MOI ratios. Ferric citrate and δ-aminolevulinic acid hydrochloride were added to a final concentration of 0.1 mM at the time of infection and 24 h after infection to compensate the low levels of endogenous heme in the insect cells. After 60 h, cells were harvested by centrifugation, washed with PBS, and microsomes of the membrane fraction prepared according to standard procedures [37]. Briefly, pellets were homogenized for 30 s in 0.1M Na/K-phosphate buffer, pH 7.4 containing 1mM EDTA and DTT and 200mM sucrose using a Fastprep (MP Biomedicals), filtered through miracloth and centrifuged for 10 min at 680 g at 4°C. The supernantant was then centrifuged for 1 h at 100,000 g at 4°C, with the pellet subsequently resuspended in 0.1M Na/K-phosphate buffer, pH 7.6 containing 1mM EDTA and DTT and 10% glycerol using a Dounce tissue grinder. P450 expression and functionality was estimated by measuring CO-difference spectra in reduced samples using a dual beam Cary 300 UV-Vis Spectrophotometer (Agilent) and scanning from 500 nm to 400 nm [37]. The protein content of samples was determined using Bradford reagent (Sigma) and bovine serum albumin as a reference following the manufacturer’s instructions.

### Ethiprole metabolism assays and LC-MS/MS analysis

Metabolism of ethiprole was assayed by incubating recombinant P450/CPR (160 μg microsomal protein / assay) or ctrl-virus/CPR microsomes in 0.1 M potassium phosphate buffer pH 7.6 with an NADPH-regenerating system (Promega; 1.3 mM NADP+, 3.3 mM glucose-6-phosphate, 3.3 mM MgCl_2_, 0.4 U/mL^-1^ glucose-6-phosphate dehydrogenase) and substrate (2 μM) at 30°C for 90 min. The total assay volume was 200 μL using three replicates for each data point. Microsomes incubated without NADPH served as a control. The assay was quenched by the addition of ice-cold acetonitrile (to 80% final concentration), centrifuged for 10 min at 3000 g and the supernatant subsequently analyzed by liquid chromatography-tandem mass spectrometry. Chromatography was performed using a Waters UPLC utilizing a Waters Acquity HSS T3 (2.1x50 mm, 1.8μm) column. Solvents were water/0.1% formic acid and acetonitrile/0.1% formic acid used in a 4 min gradient. The mass spectrometer used was a Sciex API4000 in positive ionization mode for ethiprole and its M+16 metabolite (Rt 2.97 min; m/z 412). Metabolite”quantification” is based on the area under curve and expressed as arbitrary units. Recovery rates of parent compounds using microsomal fractions without NADPH were close to 100%.

## Supporting information

S1 Fig Amino acid alignment of CYP6ER1 variants occurring in *N. lugens* lab or field populations. Key conserved P450 motifs and substrate recognition sites are annotated.
(PDF)

S2 Fig Metabolism of ethiprole by recombinantly expressed CYP6ER1vA. NADPH-dependent conversion of ethiprole to an M+16 metabolite is shown. Error bars indicate standard deviation (n = 3).
(PDF)

S3 Fig Ramachandran plot of the CYP6ER1vA model. (PDF)

S4 Fig CYP6ER1 mutant Drosophila strains: qPCR confirmation of induced overexpression of inserted genes in the F1 versus parental generations after experimental crossings. Bar graphs show mean fold-change and SEM of genes of interest relative to housekeeping genes. Asterisk’s indicate significant differences in expression (t-test, **p = < 0.05, ***p = < 0.01, ****p = < 0.001).
(PDF)

S5 Fig CYP6ER1 natural variants *Drosophila* strains: qPCR confirmation of induced overexpression of inserted genes in the F1 versus parental generations after experimental crossings. Bar graphs show mean fold-change and SEM of genes of interest relative to housekeeping genes. Asterisk’s indicate significant differences in expression (t-test, ***p = < 0.01, ****p = < 0.001).
(PDF)

S1 Table Sensitivity of transgenic strains of *Drosophila melanogaster* expressing different CYP6ER1 variants to ethiprole. Lethal Concentration 50% (LC_50_) values, and associated 95% confidence intervals (CI) are displayed for each strain derived from full dose-response bioassays. Resistance ratios (RR) are relative to the single copy variant (CYP6ER1vL) present in the lab insecticide susceptible *N*. *lugens* strain NLS or the no transgene control. *Z*-tests using compParm() function in ‘drc’ were used to compare LC_50_ values of each strain to the no transgene control line and the fly line expressing CYP6ER1vL and detect significant differences.
(PDF)

S2 Table Sensitivity of transgenic strains of *Drosophila melanogaster* expressing different CYP6ER1 variants to fipronil. Lethal Concentration 50% (LC_50_) values, and associated 95% confidence intervals (CI) are displayed for each strain derived from full dose-response bioassays. Resistance ratios (RR) are relative to the single copy variant (CYP6ER1vL) present in the lab insecticide susceptible *N*. *lugens* strain NLS or the no transgene control. *Z*-tests using compParm() function in ‘drc’ were used to compare LC_50_ values of each strain to the no transgene control line and the fly line expressing CYP6ER1vL and detect significant differences.
(PDF)

S3 Table Sensitivity of transgenic strains of *Drosophila melanogaster* expressing different CYP6ER1 variants to imidacloprid. Lethal Concentration 50% (LC_50_) values, and associated 95% confidence intervals (CI) are displayed for each strain derived from full dose-response bioassays. Resistance ratios (RR) are relative to the single copy variant (CYP6ER1vL) present in the lab insecticide susceptible *N*. *lugens* strain NLS or the no transgene control. *Z*-tests using compParm() function in ‘drc’ were used to compare LC_50_ values of each strain to the no transgene control line and the fly line expressing CYP6ER1vL and detect significant differences.
(PDF)

S4 Table Sensitivity of transgenic strains of *Drosophila melanogaster* expressing mutated CYP6ER1 variants to ethiprole. Lethal Concentration 50% (LC_50_) values, and associated 95% confidence intervals (CI), derived from full dose-response bioassays are displayed for *D*. *melanogaster* strains expressing CYP6ER1vL variants to which mutations previously implicated in imidacloprid resistance in the SRS4 and SRS5 of *CYP6ER1vA* were added. For comparison LC_50_ values derived from flies expressing the wildtype version of CYP6ER1vL in the same bioassay is shown. Resistance ratios (RR) are relative to the single copy variant (CYP6ER1vL) present in the lab insecticide susceptible *N*. *lugens* strain NLS. *Z*-tests using compParm() function in ‘drc’ were used to compare LC_50_ values of each strain to the fly line expressing CYP6ER1vL and detect significant differences.
(PDF)

S5 Table Sensitivity of transgenic strains of *Drosophila melanogaster* expressing mutated CYP6ER1 variants to ethiprole. Lethal Concentration 50% (LC_50_) values, and associated 95% confidence intervals (CI), derived from full dose-response bioassays are displayed for *D*. *melanogaster* strains expressing CYP6ER1vA to which mutations previously implicated in imidacloprid resistance were removed. For comparison LC_50_ values derived from flies expressing the wildtype version of CYP6ER1vA are shown. *Z*-tests using compParm() function in ‘drc’ were used to compare LC_50_ values of each strain to the fly line expressing CYP6ER1vA and detect significant differences.
(PDF)

S6 Table Sensitivity of transgenic strains of *Drosophila melanogaster* expressing mutated CYP6ER1 variants to ethiprole. Lethal Concentration 50% (LC_50_) values, and associated 95% confidence intervals (CI), derived from full dose-response bioassays are displayed for *D*. *melanogaster* strains expressing *CYP6ER1vL* variants to which three mutations found in *CYP6ER1vA* outside of SRS4 and SRS5 were added, or *CYP6ER1vA* to which the three mutations were removed. For comparison LC_50_ values derived from flies expressing the wildtype versions of CYP6ER1vA and CYP6ER1vL are shown. *Z*-tests using compParm() function in ‘drc’ were used to compare LC_50_ values of each strain to the fly lines expressing CYP6ER1vL and/or CYP6ER1vA and detect significant differences.
(PDF)

S7 Table Sensitivity of transgenic strains of *Drosophila melanogaster* expressing mutated CYP6ER1 variants to imidacloprid. Lethal Concentration 50% (LC_50_) values, and associated 95% confidence intervals (CI), derived from full dose-response bioassays are displayed for *D*. *melanogaster* strains expressing CYP6ER1vL variants to which mutations found in *CYP6ER1vA* inside and outside of SRS4 and SRS5 were added, or CYP6ER1vA to which the same mutations were removed. For comparison LC_50_ values derived from flies expressing the wildtype versions of CYP6ER1vA and CYP6ER1vL, or without a transgene are shown. Resistance ratios (RR) are relative to the single copy variant (CYP6ER1vL) present in the lab insecticide susceptible *N*. *lugens* strain NLS or wildtype CYP6ER1vA. *Z*-tests using compParm() function in ‘drc’ were used to compare LC_50_ values of each strain to CYP6ER1vL and detect significant differences.
(PDF)

S8 Table Sensitivity of transgenic strains of *Drosophila melanogaster* expressing different CYP6ER1 variants to compounds of intermediate halogenation between ethiprole (no halogenation) and fipronil. Lethal Concentration 50% (LC_50_) values, and associated 95% confidence intervals (CI), derived from full dose-response bioassays are displayed for *D*. *melanogaster* strains expressing CYP6ER1vL and CYP6ER1vA. *Z*-tests using compParm() function in ‘drc’ were used to compare LC_50_ values of each strain to the no transgene control line and the fly line expressing CYP6ER1vL and detect significant differences.
(PDF)

S9 Table Sequence of oligonucleotide primers used in this study. (PDF)
