# Supplementary material for: P450 gene duplication and divergence led to the evolution of dual novel functions and insecticide cross-resistance in the brown planthopper Nilaparvata lugens
Source: PLoS Genet. 2022 Jun 21;18(6):e1010279. doi: 10.1371/journal.pgen.1010279 (PMC9249207; doi:10.1371/journal.pgen.1010279)
Supplement: S1 Fig — Key conserved P450 motifs and substrate recognition sites are annotated. (PDF) [file pgen.1010279.s001.pdf]

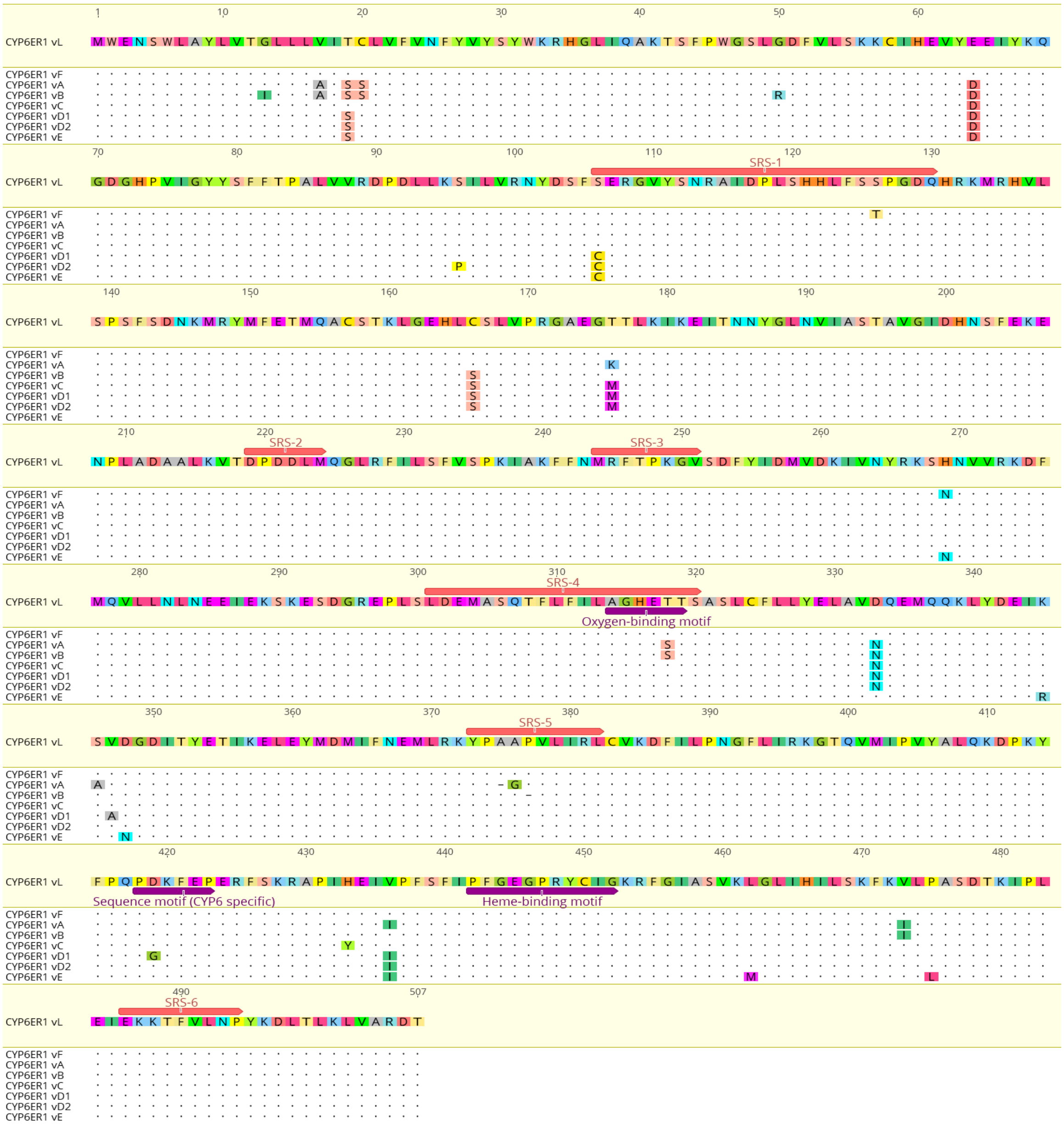

CYP6ER1 vL
MWENSWLAYLVTGLLLVITCLVFVNFYVYSYWKRHGLIQAKTSFPWGSLGDFVLSKKCIHEVYEEIYKQ
GDGHPVIGYYSFFTPALVVRDPDLLKSILVRNYDSFSERGVYSNRAIDPLSHHLFSSPGDQHRKMRHVL
SPSFSDNKMRYMFETMQACSTKLGEHLCSLVPRGAEGTTLKIKEITNNYGLNVIASTAVGIDHNSFEKE
NPLADAALKVTDPDDLMQGLRFILSFVSPKIAKFFNMRFTPKGVSDFYIDMVDKIVNYRKSHNVVRKDF
MQVLLNLNEEIEKSKESDGREPLSLDEMASQTFLFILAGHETTSASLCFLLYELAVDQEMQQKLYDEIK
SVDGDITYETIKELEYMDMIFNEMLRKYPAAPVLIRLCVKDFILPNGFLIRKGTQVMIPVYALQKDPKY
FPQPDKFEPERFSKRAPIHEIVPFSFIPFGEGPRYCIGKRFGIASVKLGLIHILSKFKVLPASDTKIPL
EIEKKTFVLNPYKDLTLKLVARDT
CYP6ER1 vF
CYP6ER1 vA
CYP6ER1 vB
CYP6ER1 vC
CYP6ER1 vD1
CYP6ER1 vD2
CYP6ER1 vE
SRS-1
SRS-2
SRS-3
SRS-4
Oxygen-binding motif
SRS-5
Sequence motif (CYP6 specific)
Heme-binding motif
SRS-6
