# Supplementary figures and images for: P450 gene duplication and divergence led to the evolution of dual novel functions and insecticide cross-resistance in the brown planthopper Nilaparvata lugens
Source: PLoS Genet. 2022 Jun 21;18(6):e1010279. doi: 10.1371/journal.pgen.1010279 (PMC9249207; doi:10.1371/journal.pgen.1010279)

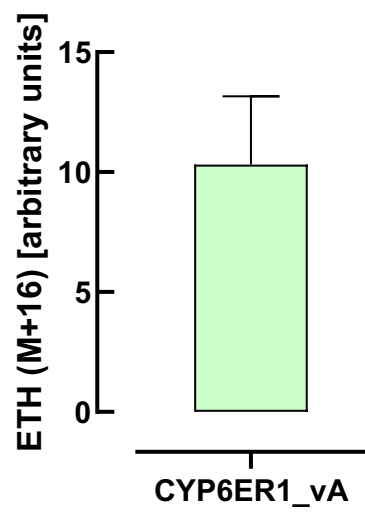

Supplement: S2 Fig — NADPH-dependent conversion of ethiprole to an M+16 metabolite is shown. Error bars indicate standard deviation (n = 3). (PDF) [file pgen.1010279.s002.pdf]

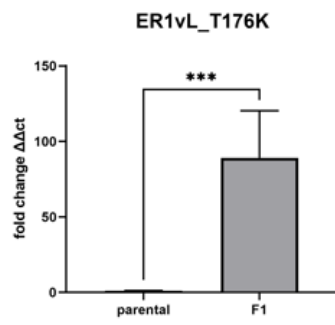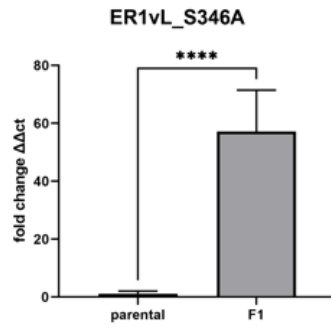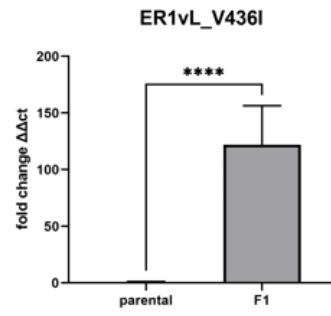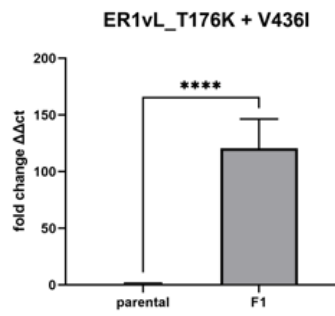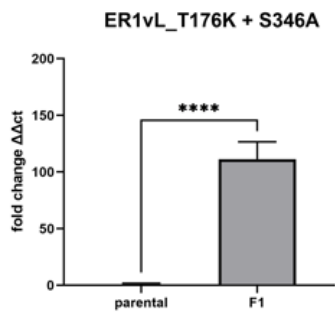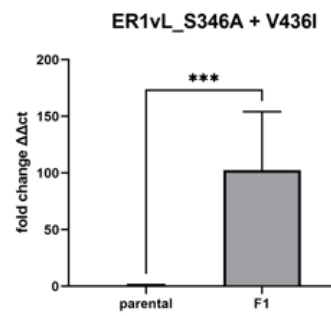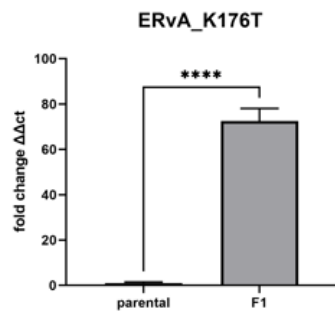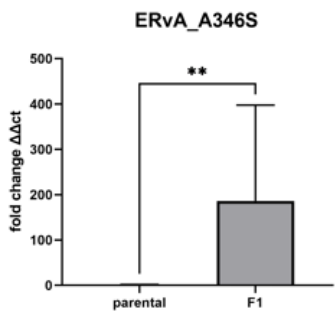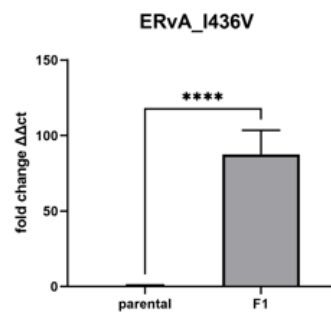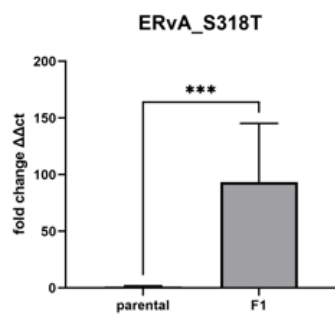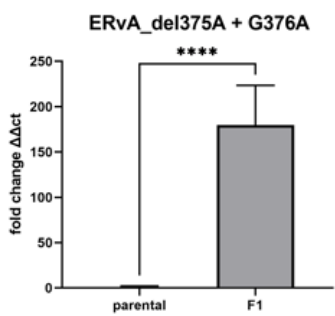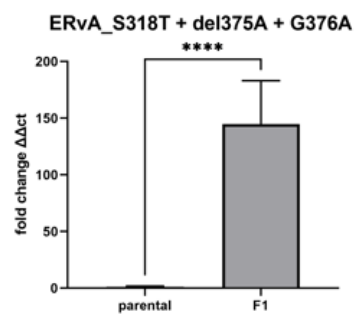

Supplement: S4 Fig — Bar graphs show mean fold-change and SEM of genes of interest relative to housekeeping genes. Asterisk’s indicate significant differences in expression (t-test, **p = < 0.05, ***p = < 0.01, ****p = < 0.001). (PDF) [file pgen.1010279.s004.pdf]

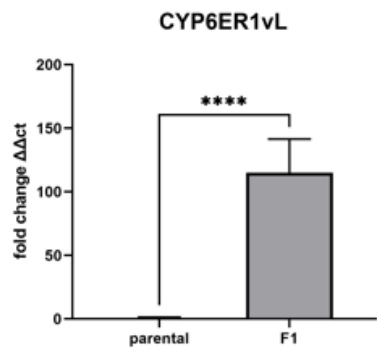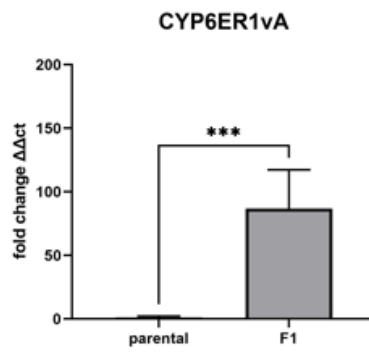

Supplement: S5 Fig — Bar graphs show mean fold-change and SEM of genes of interest relative to housekeeping genes. Asterisk’s indicate significant differences in expression (t-test, ***p = < 0.01, ****p = < 0.001). (PDF) [file pgen.1010279.s005.pdf]
