# Supplementary material for: P450 gene duplication and divergence led to the evolution of dual novel functions and insecticide cross-resistance in the brown planthopper Nilaparvata lugens
Source: PLoS Genet. 2022 Jun 21;18(6):e1010279. doi: 10.1371/journal.pgen.1010279 (PMC9249207; doi:10.1371/journal.pgen.1010279)
Supplement: S3 Fig — (PDF) [file pgen.1010279.s003.pdf]

Ramachandran Plot

saves

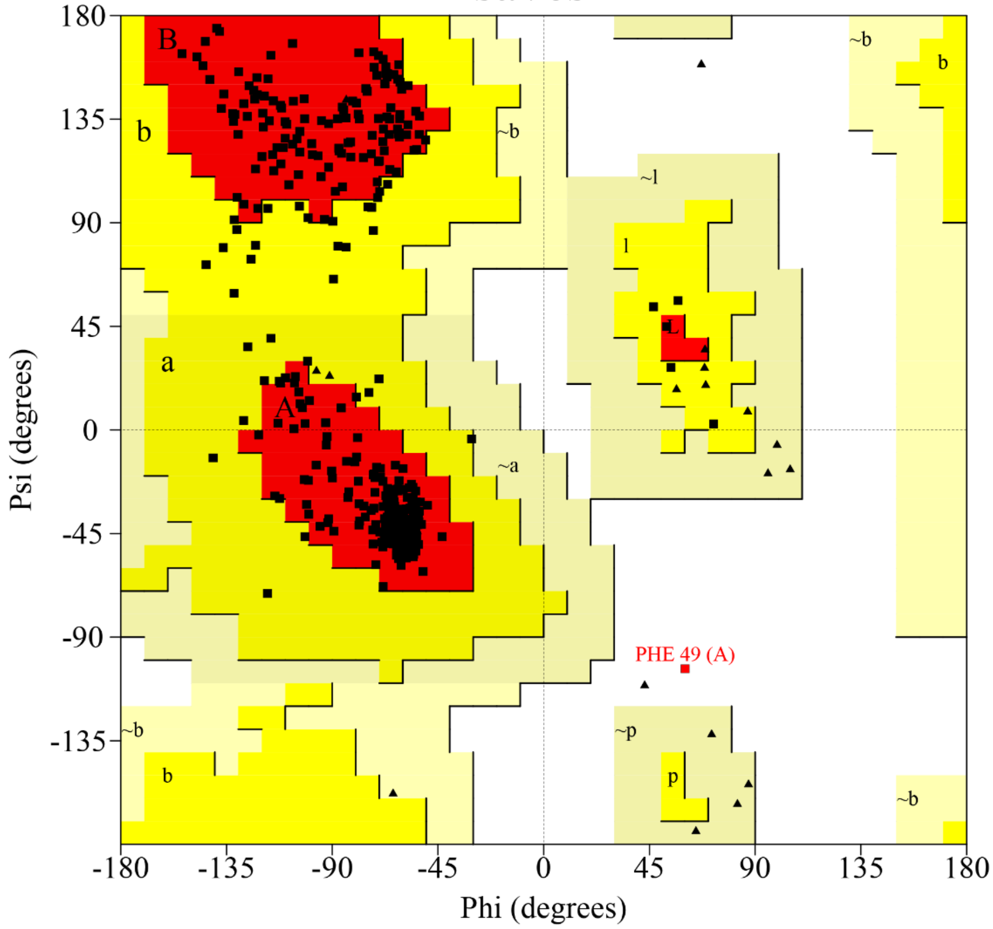

Plot statistics

Residues in most favoured regions [A,B,L]	382	91.4%
Residues in additional allowed regions [a,b,l,p]	35	8.4%
Residues in generously allowed regions [~a,~b,~l,~p]	0	0.0%
Residues in disallowed regions	1	0.2%
	----	------
Number of non-glycine and non-proline residues	418	100.0%
Number of end-residues (excl. Gly and Pro)	2	
Number of glycine residues (shown as triangles)	26	
Number of proline residues	28	

Total number of residues	474	

Based on an analysis of 118 structures of resolution of at least 2.0 Angstroms and R-factor no greater than 20%, a good quality model would be expected to have over 90% in the most favoured regions.
