# Supplementary material for: P450 gene duplication and divergence led to the evolution of dual novel functions and insecticide cross-resistance in the brown planthopper Nilaparvata lugens
Source: PLoS Genet. 2022 Jun 21;18(6):e1010279. doi: 10.1371/journal.pgen.1010279 (PMC9249207; doi:10.1371/journal.pgen.1010279)
Supplement: S2 Table — Lethal Concentration 50% (LC50) values, and associated 95% confidence intervals (CI) are displayed for each strain derived from full dose-response bioassays. Resistance ratios (RR) are relative to the single copy variant (CYP6ER1vL) present in the lab insecticide susceptible N. lugens strain NLS or the no transgene control. Z-tests using compParm() function in ‘drc’ were used to compare LC50 values of each strain to the no transgene control line and the fly line expressing CYP6ER1vL and detect significant differences. (PDF) [file pgen.1010279.s007.pdf]

**Strain**	**LC_{50} (ppm)**	**LC_{50} 95% CI**	***Comparison to No transgene***			***Comparison to vL***		
			RR	***t*-value**	***p***	**RR**	***t*-value**	***p***
**No transgene**	2.62	2.41-2.85	-	-	-	1.23	2.15	0.031*
**CYP6ER1vL**	2.12	1.77-2.54	0.81	-2.15	0.03*	-	-	-
**CYP6ER1vA**	2.93	2.71-3.16	1.12	1.80	0.07	1.38	3.36	<0.001***
**CYP6ER1vB**	2.68	2.41-2.97	1.02	0.33	0.74	1.26	2.32	0.02*
**CYP6ER1vC**	2.81	2.55-3.09	1.07	1.04	0.30	1.32	2.85	0.004**
**CYP6ER1vF**	2.17	1.93-2.45	0.83	-2.67	0.007**	1.02	0.22	0.82
