# Supplementary material for: P450 gene duplication and divergence led to the evolution of dual novel functions and insecticide cross-resistance in the brown planthopper Nilaparvata lugens
Source: PLoS Genet. 2022 Jun 21;18(6):e1010279. doi: 10.1371/journal.pgen.1010279 (PMC9249207; doi:10.1371/journal.pgen.1010279)
Supplement: S5 Table — Lethal Concentration 50% (LC50) values, and associated 95% confidence intervals (CI), derived from full dose-response bioassays are displayed for D. melanogaster strains expressing CYP6ER1vA to which mutations previously implicated in imidacloprid resistance were removed. For comparison LC50 values derived from flies expressing the wildtype version of CYP6ER1vA are shown. Z-tests using compParm() function in ‘drc’ were used to compare LC50 values of each strain to the fly line expressing CYP6ER1vA and detect significant differences. (PDF) [file pgen.1010279.s010.pdf]

Strain	LC_{50} (ppm)	LC_{50} 95% CI	RR	*t*-value	*p*-value
**CYP6ER1vA**	292.1	230.7-369.8			
**ERvA_S318T**	162.9	137.2-193.5	0.6	-3.8	<0.001***
**ERvA_del375A + G376A**	249.8	205.8-303.3	0.9	-1.0	0.3
**ERvA_S318T + del375A + G376A**	162.6	132.1-200.1	0.6	-3.5	<0.001***
