# Supplementary material for: P450 gene duplication and divergence led to the evolution of dual novel functions and insecticide cross-resistance in the brown planthopper Nilaparvata lugens
Source: PLoS Genet. 2022 Jun 21;18(6):e1010279. doi: 10.1371/journal.pgen.1010279 (PMC9249207; doi:10.1371/journal.pgen.1010279)
Supplement: S7 Table — Lethal Concentration 50% (LC50) values, and associated 95% confidence intervals (CI), derived from full dose-response bioassays are displayed for D. melanogaster strains expressing CYP6ER1vL variants to which mutations found in CYP6ER1vA inside and outside of SRS4 and SRS5 were added, or CYP6ER1vA to which the same mutations were removed. For comparison LC50 values derived from flies expressing the wildtype versions of CYP6ER1vA and CYP6ER1vL, or without a transgene are shown. Resistance ratios (RR) are relative to the single copy variant (CYP6ER1vL) present in the lab insecticide susceptible N. lugens strain NLS or wildtype CYP6ER1vA. Z-tests using compParm() function in ‘drc’ were used to compare LC50 values of each strain to CYP6ER1vL and detect significant differences. (PDF) [file pgen.1010279.s012.pdf]

Strain	LC_{50} (ppm)	LC_{50} 95% CI	*Comparison to vL*			*Comparison to vA*		
			RR	*t*	*p*	RR	*t*	*p*
**No transgene**	879.2	713.2-1083.8	1.2	1.2	0.2			
**CYP6ER1vL**	715.4	557.3-918.4	-					
**CYP6ER1vA**	3465.3	2777.9-4322.8	4.8	8.1	<0.001***			
Mutations outside SRS 4 and 5								
**ER1vL_T176K**	1908.8	1521.9-2394.0	2.7	5.3	<0.001***			
**ER1vL_S346A**	1054.2	853.6-1301.8	1.5	2.3	0.03*			
**ER1vL_V436I**	2270.4	1803.1-2858.8	3.2	6.1	<0.001***			
**ERvA_K176T**	3182.7	2546.5-3977.8	4.5	7.7	<0.001***	0.9	-0.5	0. 6
**ERvA_A346S**	2010	1600.9 - 2523.7	2.8	5.5	<0.001***	0.6	-3.5	<0.001***
**ERvA_I436V**	3730.2	2930.3-4748.4	5.2	8.2	<0.001***	1.1	0.4	0.7
Mutations within SRS 4 and 5								
**ERvA_S318T + del375A + G376A**	1110.7	860.4 - 1433.9	1.6	2.3	0.02*	0.3	-7.0	<0.001***
