# Supplementary material for: P450 gene duplication and divergence led to the evolution of dual novel functions and insecticide cross-resistance in the brown planthopper Nilaparvata lugens
Source: PLoS Genet. 2022 Jun 21;18(6):e1010279. doi: 10.1371/journal.pgen.1010279 (PMC9249207; doi:10.1371/journal.pgen.1010279)
Supplement: S9 Table — (PDF) [file pgen.1010279.s014.pdf]

Oligo name	Sequence	Purpose
CYP6ER1 vL/vF F	CATCCATGAGGTCTACGAAG	CYP6ER1 variant-specific QPCR
CYP6ER1 vL/vF R	GAGTGCTGAACAGATGGTGT	CYP6ER1 variant-specific QPCR
CYP6ER1 vA F	CTTTCTTCACCCCCGCCC	CYP6ER1 variant-specific QPCR
CYP6ER1 vA R	CCTGCATGGTCTCGAACATG	CYP6ER1 variant-specific QPCR
CYP6ER1 vB F	TCTTGTCACAATCCTGTTGCTG	CYP6ER1 variant-specific QPCR
CYP6ER1 vB R	TGGATGCATTTCTTGGACAATACG	CYP6ER1 variant-specific QPCR
CYP6ER1 vC F	GAGACTACTTCTGCATCTTTGT	CYP6ER1 variant-specific QPCR
CYP6ER1 vC R	GGAAACCATTGGGAAGAATGA	CYP6ER1 variant-specific QPCR
CYP6ER1 vD F	AGATCAAATCGGCGGATGGA	CYP6ER1 variant-specific QPCR
CYP6ER1 vD R	CGGAATCATCACTTGAGTTCC	CYP6ER1 variant-specific QPCR
CYP6ER1 vE R	CCGGAATCATTACTTGAGTTCC	CYP6ER1 variant-specific QPCR
CYP6ER1 vE F	GTATGATGAGATCAGATCTGTGA	CYP6ER1 variant-specific QPCR
Nl_Actin_F	TAACGAGAGGTTCCGTTGCC	qPCR of *N. lugens* reference gene (actin)
Nl_Actin_R	GACAGGACAGTGTTGGCGTA	qPCR of *N. lugens* reference gene (actin)
Nl_α2_tubulin_F	CCACCCTGGAACACTCTGAC	qPCR of *N. lugens* reference gene (α2_tubulin)
Nl_α2_tubulin_R	CGAAGCAGTGATCGAGGACA	qPCR of *N. lugens* reference gene (α2_tubulin)
D099 pUAST F	TCACTGGAACTAGGCTAGCA	Sequence validation of transgenic flies
D102 pUAST F	GGATCCAAGCTTGCATGCCTG	Sequence validation of transgenic flies
D100 pUAST R	AAAGGCATTCCACCACTGCT	Sequence validation of transgenic flies
D101 pUAST R	CCACCACTGCTCCCATTCAT	Sequence validation of transgenic flies
ER1vL-p2-F	TCTACAAGCAAGGTGACGGT	Testing CYP6ER1 expression in transgenic flies by QPCR
ER1vL-p2-R	ATCACCTGGACTGCTGAACA	Testing CYP6ER1 expression in transgenic flies by QPCR
UT_p1_F	GCAACAGAAGCTCTACGACG	Testing CYP6ER1 expression in transgenic flies by QPCR
T1-p1-R	GTCCTTTTGCAGTGCGTACA	Testing CYP6ER1 expression in transgenic flies by QPCR
T2-p1-R	GGGCGTACACTGGGATCATA	Testing CYP6ER1 expression in transgenic flies by QPCR
T3-p1-R	ACTTCGGGTCTTTCTGCAGA	Testing CYP6ER1 expression in transgenic flies by QPCR
Dm SDHA F	CACGACCCTCCATGATCTCG	qPCR of *D. melanogaster* reference gene (SDHA)
Dm SDHA R	CGGATGTCTCATCACCGAGG	qPCR of *D. melanogaster* reference gene (SDHA)
Dm RPL32 F	GCGCTTGTTCGATCCGTAAC	qPCR of *D. melanogaster* reference gene (RPL32)
Dm RPL32 R	GCCCAAGGGTATCGACAACA	qPCR of *D. melanogaster* reference gene (RPL32)
